# Cell size, body size and Peto’s paradox

**DOI:** 10.1186/s12862-022-02096-5

**Published:** 2022-12-13

**Authors:** Sebastian Maciak

**Affiliations:** Department of Evolutionary and Physiological Ecology, Faculty of Biology, University of Białystok, K. Ciołkowskiego 1J, 15-245 Białystok, Poland

**Keywords:** Cell size, Body mass, Carcinogenesis, Evolution, Peto’s paradox

## Abstract

Carcinogenesis is one of the leading health concerns afflicting presumably every single animal species, including humans. Currently, cancer research expands considerably beyond medicine, becoming a focus in other branches of natural science. Accumulating evidence suggests that a proportional scale of tumor deaths involves domestic and wild animals and poses economical or conservation threats to many species. Therefore, understanding the genetic and physiological mechanisms of cancer initiation and its progression is essential for our future action and contingent prevention. From this perspective, I used an evolutionary-based approach to re-evaluate the baseline for debate around Peto’s paradox. First, I review the background of information on which current understanding of Peto’s paradox and evolutionary concept of carcinogenesis have been founded. The weak points and limitations of theoretical modeling or indirect reasoning in studies based on intraspecific, comparative studies of carcinogenesis are highlighted. This is then followed by detail discussion of an effect of the body mass in cancer research and the importance of cell size in consideration of body architecture; also, I note to the ambiguity around cell size invariance hypothesis and hard data for variability of cell size across species are provided. Finally, I point to the new research area that is driving concepts to identify exact molecular mechanisms promoting the process of tumorigenesis, which in turn may provide a proximate explanation of Peto’s paradox. The novelty of the approach proposed therein lies in intraspecies testing of the effect of differentiation of cell size/number on the probability of carcinogenesis while controlling for the confounding effect of body mass/size.

**Supplementary Information:**

The online version contains supplementary material available at 10.1186/s12862-022-02096-5.

## Background

Cancer is caused by mutations in DNA leading to many cellular disorders and unregulated cell divisions. Assuming that every single cell has the same probability of developing tumorous mutations, multicellular long-lived organisms should be at higher risk of cancer initiation. However, available data failed to confirm any correlation between cancer incidence, body mass and longevity [1, 2, 3, 4, 5, 6, 8]. The lack of such a relationship was dubbed Peto’s paradox, which remains unresolved for almost 40 years and exposes our poor understanding of fundamental evolutionary mechanisms underlying the origins of cancer. Interest in Peto’s paradox resurfaced after it was postulated that its solution can provide new methods of cancer prevention and treatment for all living organisms, including humans [3, 4, 5, 6, 7, 8, 9, 10, 12]. Beyond the cognitive aspect of Peto’s paradox solution, understanding how natural selection responds to cancer can be illuminating for biomedical sciences as well. Hypothetical compensatory mechanisms driven by natural selection may include slower somatic cell turnover, redundancy of tumor suppressor genes, a more efficient immune system, better suppression of inflammation or enhanced resistance to oncogenic viruses [5, 11, 13, 14]. Therefore, debate around this phenomenon has recently arisen, suggesting many molecular and/or organismal concepts that can explain Peto’s paradox and develop new methods of cancer prevention [3, 4, 6, 9, 15, 16]. Although recent papers concerning Peto’s assumption led to wide interest in the evolution of carcinogenesis and greatly contributed to progress in the field of cancer biology, we are still far from fully understanding how potentially anticancer mechanisms operate in different organisms. Moreover, most of the emerging evidence for Peto’s paradox lacks unambiguous scrutiny and a common direction of study. As carcinogenesis, in general, constitutes a multifactorial process, any narrowed analysis devoid of required ecological context most often leads to overinterpretations and/or misguided conclusions.

One of the cardinal arguments that should be addressed here is literal interpretation of Peto’s assumptions on the key role of the basic comparison of large vs. small animals that is generally presented in the current literature [e.g., 3, 7, 11, 17]. Such an interpretation hits several constraints that preclude (at least for today) proper and honest biological reasoning. Size-induced patterns have been identified for all aspects of animal design and function from structural dimensions through life history characteristics to pharmacokinetics. Therefore, looking for the specific, mass-independent anticancer mechanisms that can be applied in most organisms along with humans seems not trivial. Another simplification goes to the assumption of invariance of size of cells that build organisms and isometric scaling of body size with the number of those. To date, it has become clear that the size of cells differs considerably at both inter- and intraspecific levels [18–20]. Thus, according to cellular architecture, the body may consist of a large number of small cells, a small number of larger cells, or a combination of both, while body mass can remain unaffected [19, 21]. However, several opinions still occur that evolutionary changes in body size are simply implications of differences only in cell number [e.g., 4–6]. A broader look on the cytological architecture of organisms, taking into account the variation, not only in number but also, in size of cells, would improve our understanding of cancer evolution and development [9]. Finally, despite the emerging constrains, looking for universal mechanism to decrease in cancer risk is still postulated, suggesting recruitment of additional tumor suppression genes or improved activation of immune system [6]. All malignant transformations and any molecular alternations accompanying them occur in a complex environment of a cell or/and whole organism. For example, any malignant transformation relies on the accumulation of multiple mutations through stepwise processes that involve several epigenetic changes and different phenotypic properties of an organism [10]. Therefore, only comprehensive analysis of selective factors related to carcinogenesis in general can be successful.

Here, I used an evolutionary-based approach to re-evaluate the baseline for debate around Peto’s paradox. I discuss in details the effect of body mass and cell size in evolutionary related cancer research. To improve a discussion concern cytological architecture of organisms, I do provide a bold analyses for variation in size of cells in birds and mammals, based on the largest ever data collection for erythrocytes area (as a proxy of cell size). Finally, I refer to broader ecological attempts in studies of carcinogenesis. The only honest discussion concerns all limitations, and possible manners of action should bring tangible and visible benefits in methods preventing the promotion and development of cancer. The novelty of the approach proposed therein lies in intraspecific testing of the effect of differentiation of cell size/number on the probability of carcinogenesis while controlling for the confounding effect of body mass/size.

## Theoretical background and paradox limitations

At first approximation, larger animals are made up of more cells. Assuming that each physiologically active and proliferating cell is at risk of malignant transformation, any evolutionary increase in the number of cells (and thus body mass) should lead to a higher cancer frequency, all else being equal. However, available data fail to support the prediction that larger animals are affected by cancer more frequently than smaller ones [e.g., 3, 6, 7]. Hence, if Peto’s paradox describes a real phenomenon, natural selection plays a very important role in the accomplishment of cancer resistance, for example, in large, long-lived animals. However, to date, evidence for changes in the mechanisms of cancer suppression between species has been scarce, while any reports remain loosely bound to each other, suggesting upregulation of cell size (CS) or cell cycle pathways. The most classic example is naked mole rats, which are characterized by a much lower mass-specific basal metabolic rate (BMR) and longer lifespan (20–30 years) than expected by their size [22], as well as simultaneous resistance to both congenital and experimentally induced carcinogenesis [23]. Although the physiology of these animals remains poorly understood, their cells exhibit upregulation of the cyclin-dependent kinase (Cdk) inhibitor p16, which prevents cell division and favors cell growth [24, 25], which is likely a mechanism of cancer prevention. Other reports suggest the occurrence of multiple additional copies of the tumor suppression gene *p53* in elephants [3, 7]. Evolutionary lowering the threshold for DNA damage by triggering p53-dependent apoptosis may constitute another common anticancer strategy. There are a few other examples, such as the resistance of human fibroblasts to malignant transformation [26] or ability to maintain telomere length and global genome integrity in rodents [17]. However, such mechanisms of cancer resistance are more of an exception than universal rules [12]. The reason behind the observed impasse may result from important limitations in studies concerning anticancer mechanisms across species. First, currently, it is impossible to collect a considerable sample size of tumor-related deaths in wild-living animals. The cancer mortality rate in humans is mostly reported as deaths per 100,000 per year, whereas, for example, Abegglen et al. [3] included 36 mammalian species in these studies, with the minimum records for each taxon set to 10. Recently published, analysis of scaling of somatic mutation rates with lifespan in mammals is based on an average of two individuals (excluding human and laboratory mice) [27]. Moreover to date, most of the studies (excluding humans) regarding cancer, in general, are based on domestic or captive individuals [see: 3, 12 or 27], which are much less sensitive to environmental factors (physical activity, predatory pressure, natural pathogens, reproduction, etc.), that are cardinals for natural selection. Even if we could overcome these constraints, the challenging question remains: does a single molecular observation always translate into a functional change producing specific anticancer mechanisms? That is why, the great majority of reports on Peto’s paradox use theoretical modeling or indirect reasoning mostly based on interspecific comparative (and therefore correlative) inference [e.g., 4, 6, 10, 13]. However, to make any progress in the field, experimental studies are needed.

## Confounding effect of body mass

**Fig. 1:**
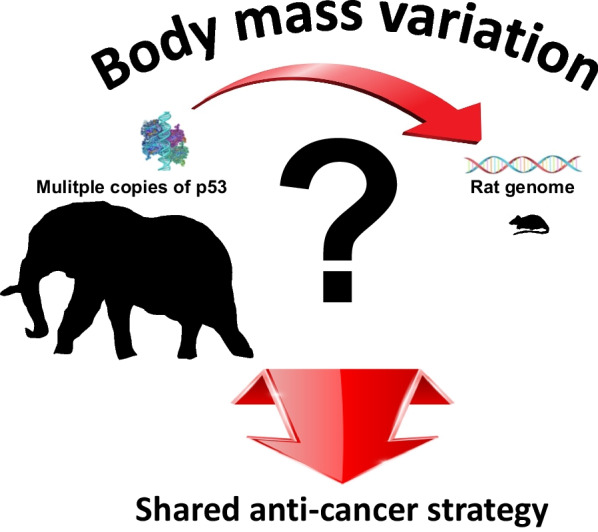
Observed variation in body mass via organismal traits that are by-products of body mass diversification (i.e. energy expenditure rate, cellular architecture, growth rate, efficiency of defensive mechanisms, longevity) may translate to a common, species-specific anti-cancer strategy. However, can we share tumor suppression mechanisms found, for example, in elephants (multiple copies of p53 protein) into genome of a rat without affecting its other physiological pathways?

Even though, recently, a few papers have suggested turning to intraspecific analyses based on chosen phenotypic traits [i.e., 5, 9], most of the statements presented in the current literature pointed out the key role of the basic comparison of large vs. small animals [e.g., 3, 6–8, 11, 17]. Body mass constitutes a basic biological trait, and no single factor is more dominant in constraining animal design. As animals display dramatically different body masses, a transfer of anticancer mechanisms between phylogenetically distant species seems impossible (Fig. 1). Therefore, it is necessary to underline that explaining the changes in cancer suppression mechanisms by comparing species is unreasonable because evolution proceeds at the population level (i.e., within-species). Consequently, species differ with respect to the mechanisms of cancer suppression as they evolve independently of each other. For example, it would be naive to expect that potential mechanisms of cancer suppression found in elephants are the same as those, operating, for example, in naked mole rats. Moreover, the body mass itself has such an overwhelming influence on the evolution of organisms, that comparing large and small animals is fundamentally impossible due to a multitude of factors. Hence, the crux of Peto’s paradox does not lie in body mass per se. Rather, it is directly related to the other biological factors as suggested earlier, e.g., cell number, cell size and cell specific metabolic rate (which adds to whole body energy expenditure). This supposition is strongly supported by the theoretical model of carcinogenesis presented in the paper of Maciak and Michalak [9], which showed that relatively minor yet cardinally important differences between the cell sizes of small and large animals are sufficient to explain the lack of a proportional increase in the risk of malignant transformation with increasing size of organisms. Such a perspective exposes the critically important difficulty with understanding Peto’s paradox: its explanation does not need to be based on comparative analyses of large vs. small organisms, as claimed elsewhere. Rather, the test of Peto’s paradox should hinge on comparisons of equal sized organisms, yet differing with respect to the specific biological traits. Therefore, one of the promising approach to tackle the paradox is to find a common denominator for the major factors, that is, to examine the effect of certain anticancer mechanisms while controlling for body mass.

### Intraspecies comparisons as a solution to Peto’s paradox

Broad between-species comparisons are willingly adopted by scholars of Peto’s paradox mostly to take advantage of a wide range of interspecific variations, enhancing statistical power. However, several studies have shown that a within-species perspective can also be informative. For example, accumulating evidence suggests that intraspecific variation in BMR or in its specific components (e.g., hormone levels) constitutes an important risk factor for most cancers [5, 6, 28]. Such hypothesis may be based on the well-established relationship between total body BMR and body size. An example is the changes in human height. According to the so-called multistage model, the hazard ratio for overall cancer risk per 10 cm-increase in human height is approximately 1.1 [5]. A positive correlation between body size and cancer incidence has also been shown in studies concerning cancer risk in tall women [29, 30]. Noble et al. concluded that 50% of the variation in cancer risk is due to tissue size (larger tissues are more prone to cancer development than smaller tissues) independent of stem cell division number. Similar to humans, large dog breeds exhibit a higher incidence of osteosarcomas and other carcinogenic mutations [31]. Such results strongly suggest that understanding the basis of this relationship could lead to new methods of cancer prevention. If the increase in organismal body size promotes tumor development, then—just in agreement with the overall concept of Peto’s paradox—low height (and small size) should result in a reduced chance for malignant transformation as well as other metabolic-related disorders. Consistent with this idea, studies on Ecuadorian populations of human dwarfs have shown only one nonlethal malignancy and no cases of diabetes during the 22 years of monitoring [32]. As genetic analysis of Ecuadorian subjects revealed mutation in the growth hormone receptor gene leading to severe growth hormone receptor (GHR) and IGF-I deficiencies, it was suggested that reduced expression of pro-growth specific genes protects against cancer and favors lifespan extension [32]. Indeed, reduced activation of growth-promoting pathways in GHR knockout mice, as well as Ames dwarf and Snell dwarf mice lacking GH, resulted in greater stress resistance, reduced inflammation, increased reservoirs of pluripotent stem cells, and extended longevity [33]. These results support the idea that defects in metabolic-sensing pathways, such as IGF, encourage cells to protect themselves and their DNA rather than to grow and divide, which is a starting point in carcinogenesis. However, it remains unclear whether blocking chosen molecular tracks could protect against metabolic-related diseases without affecting other, not less important, components of the cellular machinery. Long-term analyses of human achondroplasia (the most common dwarfism condition) showed that the overall mortality and age-specific mortality at all ages remained significantly increased as a result of serious neurological and heart-related diseases compared to the general population [34]. In the case of an Ecuadorian population of human dwarfs, only individuals aged 10 or older were included in the study due to high mortality from metabolic disorders during childhood [32]. Although animal models for dwarfism (e.g., GHR-KO mice) strongly suggest that deletion of GH signals leads to extension of longevity, those animals are hypothyroid, hypothermic and have reduced spontaneous physical activity [33]. Such phenotypic effects are usually deadly outside of laboratory conditions, and individuals with mutations in growth hormone receptors are rarely seen in natural environments. Nevertheless, to date, increasing evidence strongly suggests that pathways that normally regulate key biological traits, such as growth and metabolism, also promote aging and genomic instability. Thus, there is no doubt that the relationship between the metabolic properties of an organism and cancer risk exists and is likely a multifactor phenomenon [9, 35].

Even though the metabolic rate is a function of body size, the full explanation for the relationship between the size of an organism and carcinogenesis will remain elusive unless all of its components are taken into consideration. Nunney [5] noted, that the multistage model for carcinogenesis leads to the prediction that (1) larger individuals within a species are more prone to cancer due to their greater cell number but (2) that larger species will generally have no such tendency due to their evolutionarily enhanced levels of cancer suppression. This evolutionary resolution of Peto’s paradox (prediction 2) is expected to be true only if the underlying assumption of the model (prediction 1) is correct. However, Cagan et al. [27] suggest that not total cell number, but rather metabolism related general organ dysfunction contribute to both cancer and other common diseases. Therefore, enhanced mechanisms of tumor suppression may not be related to body mass/size or to differences in the number of cells alone, but rather to the cell size variation and/or, consequently, cell-specific metabolic rates [9, 10, 16, 27]. Such an approach may improve our understanding of ideas underlying Peto’s paradox and simultaneously allow a number of inconsistencies to bind with erroneous assumptions of cell size fixity.

## Cell size variation and its implication for carcinogenesis

**Fig. 2:**
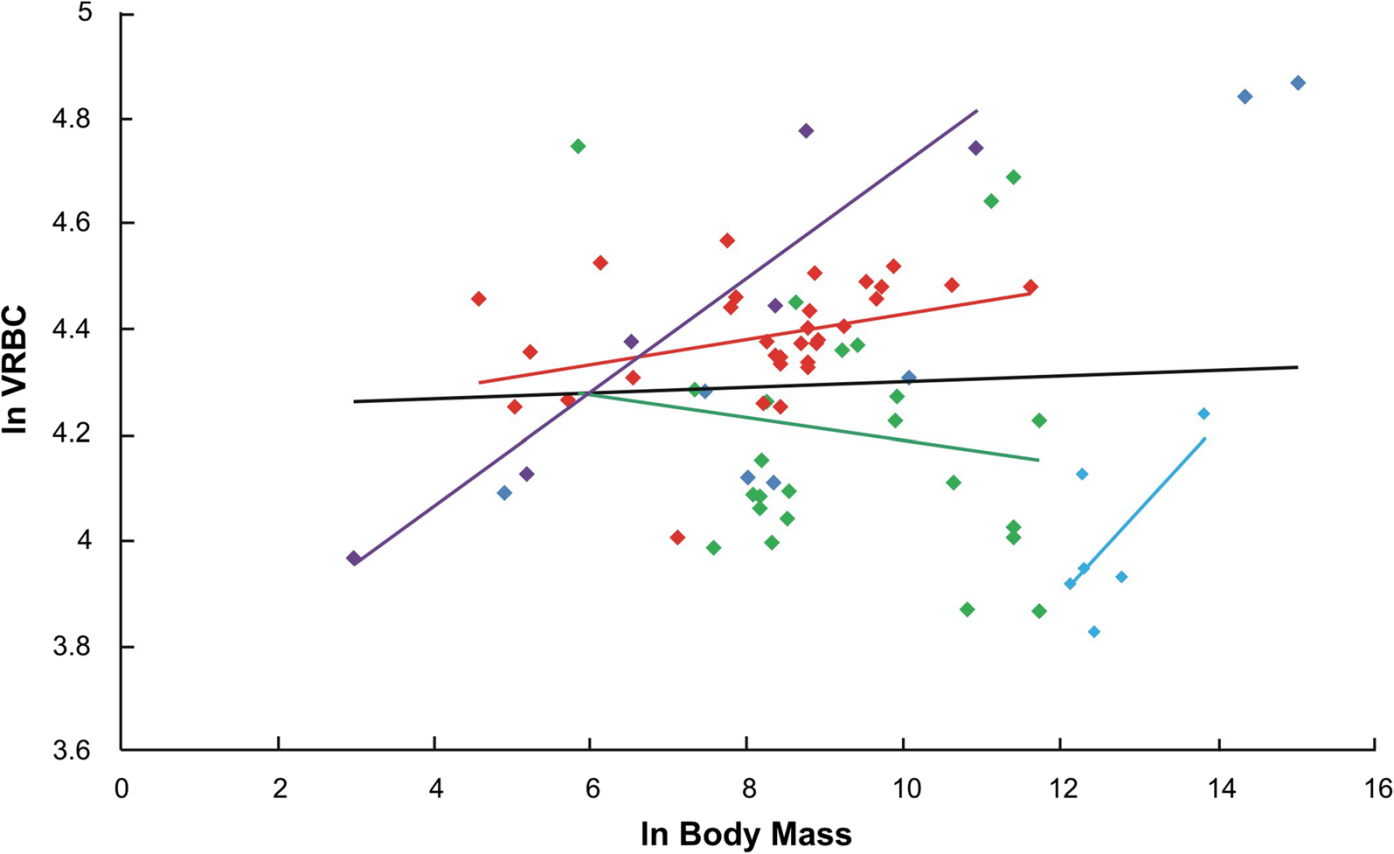
Plot of the logarithm of red blood cells volume (VRBC) versus the logarithm of body mass in mammals after Savage et al. [37]. 95% CI of the slope for all 74 records (black line) falls around the value of 0, indicating lack of relationship between cell size and body mass in mammals. However, analyses conducted on orders level revealed heterogeneity of the slopes (p < 0.001) pointing to strong relation of size of cells with body mass at lower taxonomical levels; Slops for n ≥ 6 records are shown only: *Carnivora* (green), *Primates* (red), *Perrsiodactyla* (blue), *Rodentia* (purple); remain records (dark blue)

**Fig. 3:**
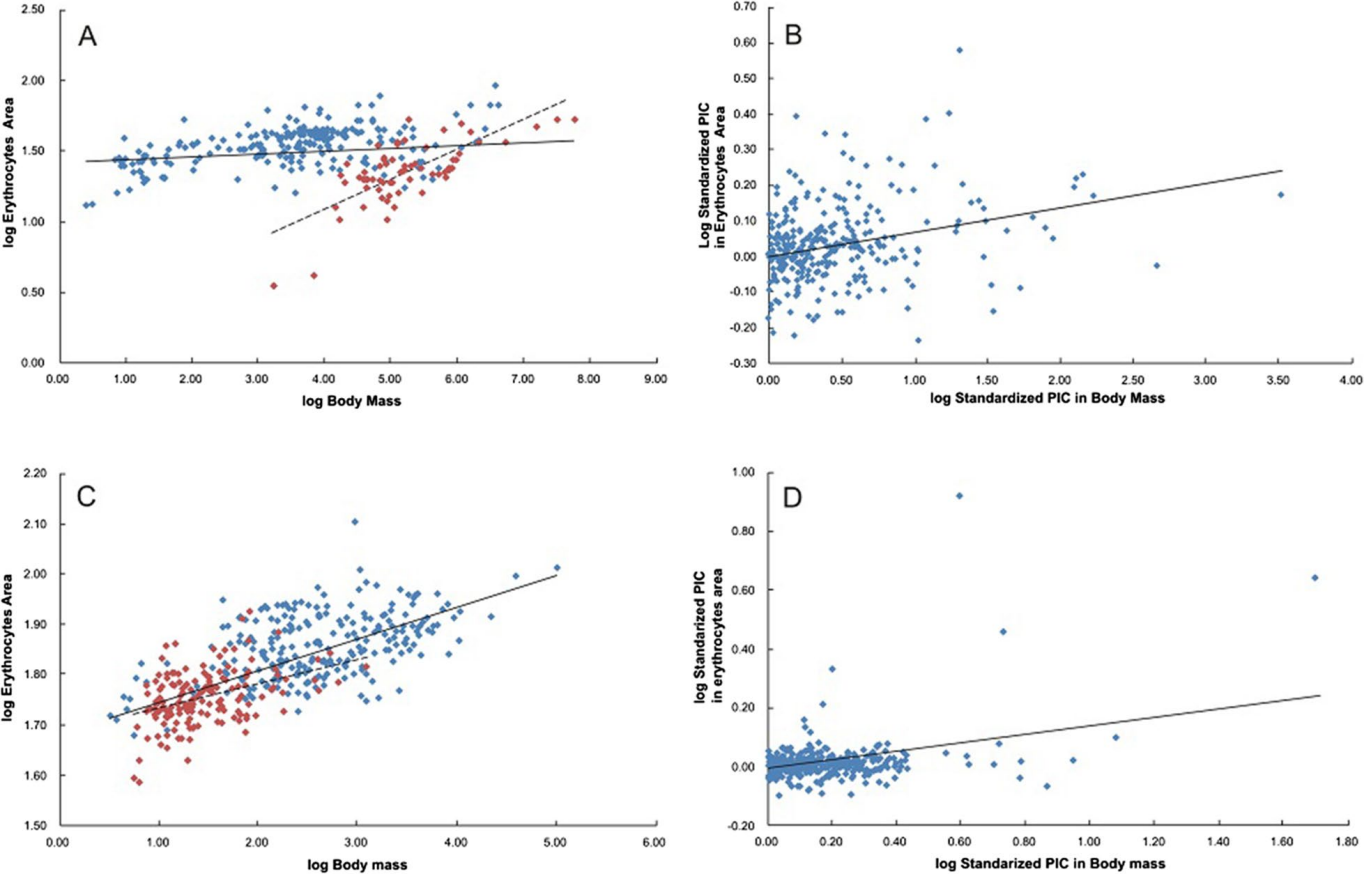
Plots of the logarithm of erythrocytes area versus the logarithm of body mass and standardized phylogenetically independent contrasts (PIC) for this relation in mammals and birds. The size of erythrocytes significantly depends on the body mass in mammals, both in conventional (**A**) and phylogeny-corrected (**B**) analyses (n = 298, p = 0.01 and n = 297, p < 0.0001, respectively). Analogous relation is observed for birds; classic (**C**) and phylogeny-corrected (**D**) correlation plot (n = 393, p < 0.0001, n = 364, p < 0.0001, respectively). In both analyzes, the exponent values are close, albeit different from 0 (mammals and birds conventional method respectively 0.019 ± 0.0074, 0.0629 ± 0.0033 and phylogenetically informed methods, respectively 0.062 ± 0.0096, 0.1720 ± 0.0181). The analysis of the slopes among 8 orders of mammals (for n ≥ 7 species in each order) and 12 orders of birds (for n ≥ 12 species in each order) showed that these relationships are strongly heterogenic (mammals: F_1.7_ = 20.5374, p < 0.0001; birds: F_1.11_ = 3.608, p < 0.0001); see Additional files 2 and 3. Chosen orders: *Artiodactyla* for mammals and *Passeriformes* for birds are shown (red rhombs)

One of the cardinal misunderstanding of Peto's paradox is followed by suggestion that evolutionary changes in body mass of organisms are simply implications of changes in cell number only [e.g., 5, 6, 12, 36]. Such reasoning is mostly based on work of Savage et al. [37] postulated size invariance of most types of cells including erythrocytes, hepatocytes, or epithelial cells. Even though, we will consider the original data of Savage et al. [37], the correction of those for phylogeny shows the considerable heterogeneity of the slopes at orders level (Fig. 2). It suggests that the potential variation in CS may be masked by too little number of records analyzed (for example, only 74 species for erythrocytes area in study of Savage et al. [37], Additional file 1) across phylogenetically closely-related species. Interestingly, extended study on size of erythrocytes (393 and 298 records, for birds and mammals respectively, including over 20 phylogenetically distant orders; see Additional files 2 and 3) revealed a strong positive correlation between area of those cells (here used as a proxy of CS) and body size (Fig. 3A, B). This relation holds also when correction for phylogenetic signal is applied (Fig. 3B, D; see Additional file 4), verifying the evolutionary pattern -at least in homeotherms- of increasing in size of cells with body size enlargement. Undoubtedly, erythrocytes constitute a distinct type of cells in context of their morphology (considerably smaller, easily deformable, unnucleated in mammals) or cancer potential (non-proliferating, low metabolism). Nevertheless, the size of red blood cells is strongly correlated with the volume of other cell types in teleost fishes [38], birds and amphibians, but poorly in mammals having nucleus-less erythrocytes [18], suggesting that epigenetic mechanisms determining positive CS relationships in tissues are conserved. Moreover, the number of records available for the size of erythrocytes is several times greater than for any other tissue, making blood cells reasonable choice in comparative and evolutionary studies. However, variation in size of cells is observed also for other tissues in both, body mass-depended manner among various taxa [18] and irrespective of body mass on intraspecies level [19]. As strength and course of the relation between size of cells and body mass may vary at different taxonomical levels (for example, this relation is negative in order of shorebirds, *Charadriiformes;* see Additional file 3), any adjustment in CS should be considered in the phylogenetically context, especially in closely related species.

**Fig. 4:**
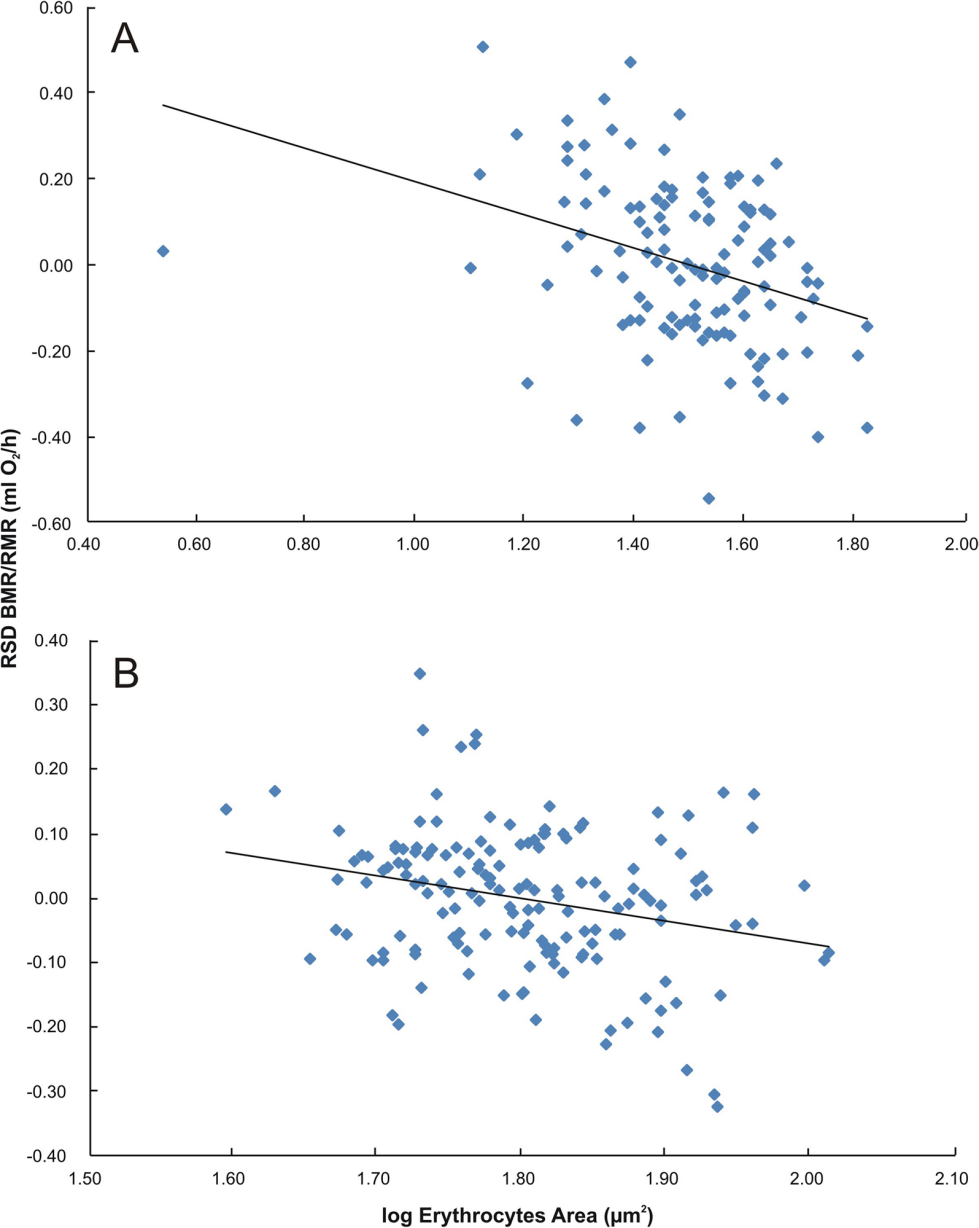
Relationship between residual values of basal metabolic rate (RSD BMR) /or resting metabolic rate (RSD RMR) and erythrocytes area in mammals (**A**) and birds (**B**); (n = 122, p = 0.0001, a = (− 0.388 ± 0.098) and n = 153, p = 0.0013, a = (− 0.347 ± 0.138) for mammals and birds respectively)

Currently, many studies refer to differences and adaptive changes in the tissue-specific size of cells as one of the most prominent features among various organisms [e.g., 9, 10, 16, 19, 39, 40, 41]. For example, erythrocytes area strongly correlates with body mass-corrected basal metabolic rate in both, birds and mammals (Fig. 4; see Additional file 1). That is why, widely observed cell size variation on both intra- or interspecies levels and its implication for organisms functioning cannot be ignored [9, 20]. In fact, cell size, cell number and cellular/organismal metabolism are jointly regulated by common signaling pathways, such as PI3K/Akt/mTOR, Myc, or Hippo-YAP [16, 43]. These regulatory mechanisms are frequently deregulated during tumorigenesis, resulting in wide variations in cell sizes and increased proliferation in cancer cells [e.g. 10, 44]. Thus, the evolution of metabolic rates, cell sizes and predisposition to malignant transformation should not be considered separately. To date, it is generally known that cell size has a direct impact on many physiological traits, including those related to cancer. First, CS homeostasis is dynamic system linking cell growth and cell division rate [10, 16, 41, 42, 45]. An increase in the division number of small cells (e.g. stem cells) may simply translate into higher cancer incidence due to errors in DNA copying with each single cell multiplication [10, 16, 27, 46, 47]. Second, the size of cells is one of the determinants of the cellular metabolic rate and costs of maintenance of the membrane gradients [42]. Consistent with the Cell Metabolism Hypothesis [21], smaller cells maintain higher metabolic rates due to a greater surface-to-volume ratio [e.g., 20, 38, 40, 48]. Postulated increase in the rate of cellular metabolism is usually related to higher production of reactive oxygen species (ROS), directly translating into chances for tumor initiation [9]. From the cancer perspective, evolutionary decrease in cell size should then bring twice the disadvantages: (1) quickly dividing, small cells should be especially prone to tumor development, while (2) their high-energy turnover (and therefore high ROS production) should considerably increase the chance for cancer initiation. However, emerging evidence suggests that a negative relation between CS and metabolic rate is not a common rule, and exceptions can be easily found. For example, cell size itself can be a limiting factor to the rate of cellular metabolism, especially in specialized cells (such as hepatocytes) involved in the multitude of concurrent metabolic processes. It has been proposed that increase in size of cells characterized by extremely high metabolic rates, improve deflection of the limiting effect of molecular crowding [19, 20]. The increase in the function of these cell lines involves other cellular processes related to the synthesis and excretion of biomolecules [10, 19]. In such cells, increased demand for energy supply may be adjusted by changes in mitochondrial number and/or mitochondrial connectivity. The expansion of intracellular network made of these organelles enable improved metabolites and protons transportation what can overcome any size-depended metabolic limitations [42]. Nonetheless, molecular crowding increases the efficiency and thermodynamic activity of protein synthesis machinery but also obstructs the diffusion of molecules within the cells. Metabolically active tissues made of small cells may not be able then to perform high level of activity because biochemical reactions in their small-volume cytoplasm are more prone to the dumping effect of molecular crowding [19]. The high concentration of cellular nutrients in metabolically active tissues (especially glucose and proteins in hepatocytes or enterocytes) usually leads to the activation of the *mTOR* gene by the specific serine-threonine kinase Akt [44, 49, 50]. mTOR promotes the cytoplasmic accumulation of Rim15 kinase (and thus its inhibition) responsible for the progression of cells through mitotic phase S [49]. The resulting intensive protein synthesis and inhibition of the cells in G1 phase point to an increase in cell size. Similarly, in mice, overexpression of tNOX (a cancer-specific and growth-related cell surface protein) leads to an average 20% increase in the size of cells compared to wild-type individuals [51]. Therefore, in certain cases, environmental activation of specific molecular pathways forces the cells to be larger than normal. However, the above-described cardinal effect of cell size as a main driver of Peto’s paradox still lacks experimental scrutiny.

### Evolutionary approach to carcinogenesis

**Fig. 5:**
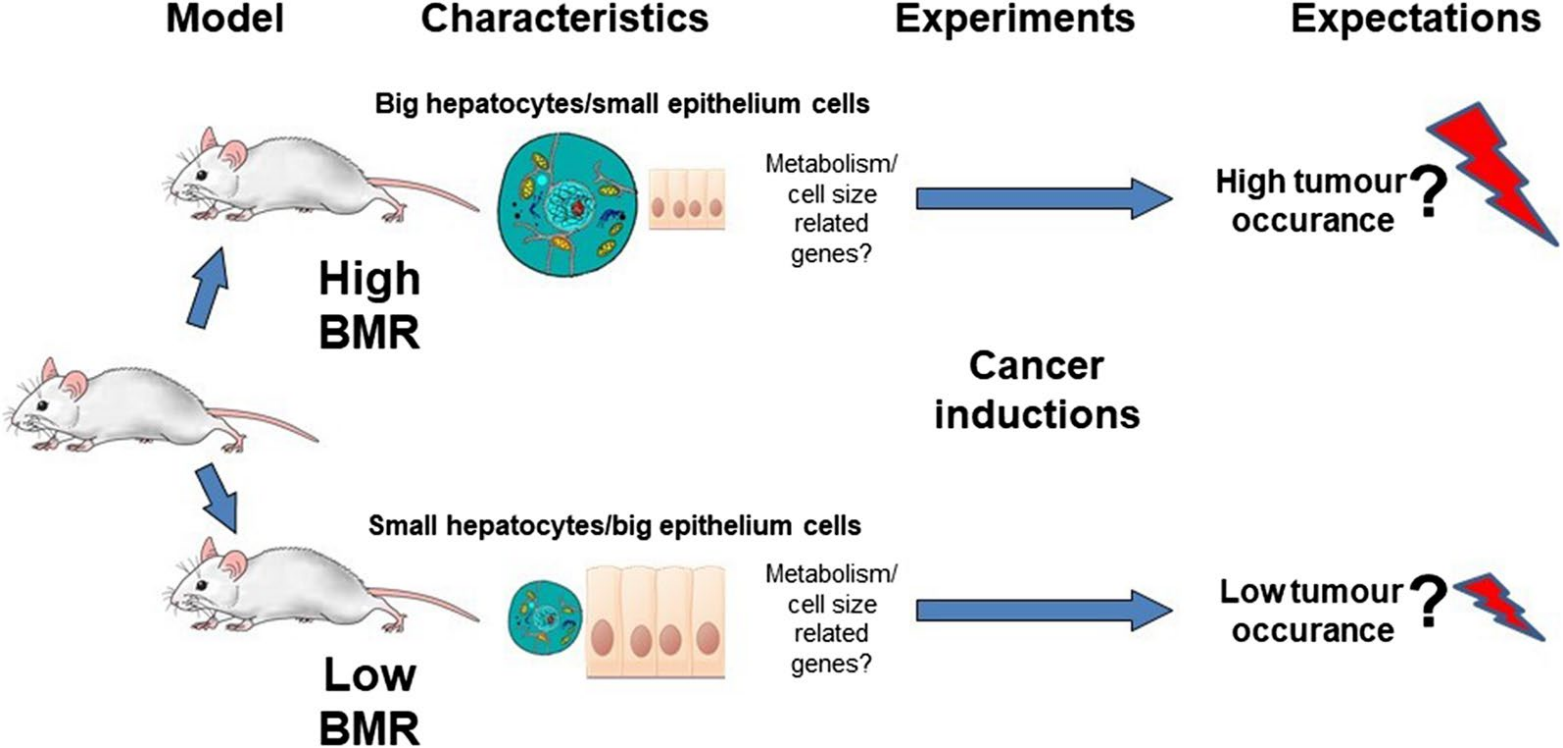
Exemplary research model to test the role of organismal metabolism and cell size in cancer development. Animals originate from the artificially selected lines for the high and low basal metabolic rate (BMR) can be found to be a cellular chimeras with considerable variation in cell size between metabolic lines

Evolution of cell size is another underappreciated key factor underlying carcinogenesis in both direct and indirect ways, which must be investigated thoroughly. Theoretically, there is little doubt that an evolutionary trade-off exists between CS, the rate of metabolism, and the ability to sustain effective defense mechanisms. Several studies indicate that any shift in average rate of energy expenditure may constitute a significant risk factor for many diseases, including cancer [27, 35, 43, 52]. For example, the intoxication rate and overall immunological activation are strongly correlated with BMR [53, 54; see Table 1]. Similarly, the high-energy turnover is also associated with lipid membrane peroxidation, negatively affecting multiple cellular functions [55]. In general, most of the cellular aerobic pathways lead to the formation of reactive oxygen species. These in turn, in higher concentrations, oxidize biological membranes, proteins, and nucleic acids resulting in misfunction of those biomolecules, cell ageing, and carcinogenesis [9, 28, 35]. As the rate of ROS production in a cell is a function of basal metabolic rate [2, 9], increased BMR can negatively affect organismal physiology via metabolism-related mechanisms, leading to formation of genetic mutations, and ultimately cancer. Although, a general relation between energy expenditures and probability for malignant transformation seems well established, for any further practical consideration, direct empirical studies are needed. Unfortunately, in practice, looking for explanations of Peto’s paradox is considerably difficult, as most species are characterized by natural variation in body mass influencing both metabolic rate and CS [20]. Therefore, solution to this problem should be provided by experiments based on intraspecific models of similar size where traits directly related to carcinogenesis can be easily controlled (Fig. 5). Such systems should emulate mechanisms of natural selection and thus markedly depart from the research paradigm of simplified animal models having knocked-out or overexpressed genes widely adopted in oncology and bearing unclear relations to the evolutionary context of carcinogenesis. Although single gene manipulation has greatly contributed to considerable progress in the field of genetics and cell biology, a deeper understanding of tumor initiation and its progression requires an evolutionary approach [36, 47, 56]. Genetically manipulated models disregard combinations of allelic variants and interactions between multiple loci, which significantly affect anticancer mechanisms molded by natural selection [36]. These shortcomings gave rise to debate on the evolutionary context of carcinogenesis and its possible contribution to understanding the mechanisms of cancer (e.g.: [8, 9, 36, 38, 56, 57]. Today, the concept that almost all neoplasms consist of highly diverse populations of cells that evolve via somatic evolution is well established [10, 27]. Moreover, the fitness of the cells, similar to the fitness of an individual organism, depends not only on their genomic sequences and protein expression but also on their interactions within the complex microenvironment of the body [36]. This is the most parsimonious evolutionary reason why most genome-wide association studies fail to explain more than a small percentage of the variation in any trait, including cancer. Hence, to deepen our understanding of carcinogenesis, it is essential to perform studies at the organismal level, allowing for comprehensive analyses of the within-species variation of complexes of traits related to cancer. In particular, a strong test of the associations between metabolic rates and probability of cancer may be provided by artificial selection experiments emulating the action of natural selection. Such experiments allow for manipulation of allele frequencies directly related to the expected associations while leaving other frequencies unchanged. To date, artificially selected animal models have been used to comprehensively and successfully investigate specific traits as, for example: body mass [58, 59], cell area [60], basal metabolic rate [61], or aerobic metabolism [62]. Moreover, such animals often display diversification in others, selection-related traits that can broaden our view on investigated issue. For instance, the lines of laboratory mice that had been selected for high and low BMR, were also recognized as cellular chimeras with the metabolism-related cells (hepatocytes, kidney proximal tubule cell, and duodenum enterocytes) considerably smaller and erythrocytes and skin epithelium cells substantially larger in individuals characterized by low energy expenditure rate [19]. Interestingly, apart from the variation in BMR and CS such lines differ distinctly with respect to the other key traits directly related to carcinogenesis that are summarized in Table 1, while notably, their body mass remain fixed.

**Table 1:** Summary of BMR-dependent traits that may influence the carcinogenesis process in mice characterized by a high basal metabolic rate

BMR correlated trait	Trait response	Statistical reliability	References
Mass of metabolically active organs	Increase	P < 0.0001; (n = 40)	[63]
Hepatocyte size	Increase	P = 0.002; (n = 15)	[19]
Skin epithelium cell size	Decrease	P = 0.007; (n = 15)	[19]
Immune response (KLH)	Decrease	P < 0.01; (n = 26)	[54]
Oxidative damage	Increase	P < 0.01; (n = 20)	[55]
Heavy metal intoxicate	Increase	P < 0.001; (n = 7)	[53]
Hepatocyte mTOR expression	Increase	P < 0.001; (n = 20)	[43]
Colorectal cancer development	Increase	P < 0.001; (n = 12)	(Unpublished data)

*KLH* Keyhole Limpet Haemocyanin, *mTOR* mechanistic Target of Rapamycin gene

Such an exemplary organism model offers then a consensus on the most effective phenotypic traits for the study of the various aspects of carcinogenesis with generally unlimited number of repeats to ensure adequate statistical power. Observed between-line variation of cellular architecture as well as differentiation of BMR should allow then to cross-check for the contribution of cell size, cell division rates, and whole-body metabolism on the probability of initiating cancerous changes. It would be therefore critical to clarify, when the cell-specific metabolic rate is directly related to the cell size, whether small cells with a higher division rate have a greater risk of cancer initiation than bigger slowly dividing cells (Fig. 5)? However, when the cell-specific metabolic rate is mainly driven by processes other than those directly related to CS (such as molecular crowding), are larger but more metabolically active cells more likely to develop cancer than smaller cells with a lower energy turnover rate (Fig. 5)? Nevertheless, a high metabolic rate seems to be linked to cancer either through the small CS (because small cells have a higher division rate) or large cell size (because of molecular crowding in metabolically active cells, such as hepatocytes). Thus, widely observed variation in the rate of metabolism which translates to physiological but also to anatomical differentiation, seems to offer important initiative to form a consensus for testing putative cellular mechanisms underlying Peto’s paradox.

Variation in traits that may be related to cancer (e.g. above mentioned energy expenditure rate or immune system efficiency) is also observed in natural populations [61]. However, unlike to model animals, experiments based on wild species are usually characterized by small sample size and/or additional variation in body mass which hamper the power of statistical analysis. However despite the few limitations, cancer related within-species studies become increasingly attractive thanks to the wealth of information that are applicable for broader evolutionary inference [5, 9, 10, 57]. The control for the impact of body mass on specific physiological traits may be achieved by appropriate means (e.g. ANCOVA with whole-body mass as a covariate), which effectively accounts for the effect of mass differences and have become widely accepted by integrative physiologists [61].

### Molecular targets for cell size-dependent carcinogenesis

Cell size itself depends mainly on extracellular growth factors, nutrient availability, and/or nuclear regulatory genes [39, 40, 45]. All of these factors impinge on three main points of control occurring independently at the cellular, organ, and organismal levels through specific signaling pathways. Changes in the expression and/or dysfunction of genes involved in CS control at any level of biological organization have also been reported in a wide variety of human cancers [e.g., 5, 10, 16, 45]. Molecular insight into carcinogenesis is urgently needed, as potential cancer resistance is most likely a complex adaptation standing on at least few (not single) mutation(s) [12]. Based on the notion of a multistage model of carcinogenesis [cf. 4, 5, 10], potential differences in cancer propensity arise due to mutations in the key genes affecting their expression. Genes dysfunction may be additionally enhance by chromosomal instability (changes in chromosomal number and/or its structure) usually result from erroneous DNA replication or repair [47]. However today, remaining challenge in any cancer studies is to determine the main drivers responsible for organism malignancy, which concomitantly stand as a promising targets for a future therapeutic action [64]. According to present knowledge, the key component of cellular growth falls in the Akt/mTOR signaling pathway. mTOR protein integrates intracellular signaling pathways in response to changes in the amount of available nutrients (particularly glucose), oxygen or cellular energy levels [e.g., 49, 50]. A high concentration of mTOR is also observed in many types of cancers, suggesting that increased expression of the *mTOR* gene may be one of the important factors supporting tumor development [16, 44, 50, 65]. However, besides the Akt/mTOR signaling pathway, CS may depend on other molecular components, which have been shown to significantly affect the cell cycle, metabolism and carcinogenesis. Most of these factors are centered around so-called extracellular growth factors, such as GH, IGF, or PDGFα (platelet-derived growth factor α) [16, 44, 66], or intracellular regulators of the cell size/cell division rate, such as *p27*, *p53*, or *Ras/myc* genes [10, 67, 68]. Proposed genes and their molecular pathways constitute an exemplary list of targets that may represent natural mechanisms of cancer resistance and should be investigated thoroughly. Any changes in the activity of specific components and their products involved in both cell size and cancer-related processes should translate directly into respective susceptibilities to tumorigenesis. The comprehensive study of the key elements representing size control signaling pathways at the inter- and intracellular levels will improve our understanding of cell growth and cellular metabolism in relation to carcinogenesis.

## Conclusions

To make any further progress in cancer biology, the current approach needs to be re-evaluated. Although, the assumptions underlying the Peto’s paradox are clear and reflected in empirical data [e.g. 8], the idea behind this phenomenon is not to confirm its existence or not. It is rather to highlight the key role of life-history evolution in shaping natural anti-cancer defences and provide most promising mechanisms for future clinical interventions. To date, broadly suggested between-species comparisons cannot bring decisive results due to substantive limitations and/or multithreading of proposed explanations. At this moment, the consideration of Peto’s paradox need for distinguish between observations concern cancer incidence across species (small vs. large comparisons) and analyses of specific mechanisms exist on cellular level, which do not require consideration of body size. The viable approach to tackle this phenomenon and the evolution of cancer may follows by intraspecies studies based on the variation in traits directly related to carcinogenesis. Any cancer type can be considered a dysfunction of cellular metabolism, cellular growth, or a combination of both. Thus, it is vital to clarify here that organismal metabolism may be linked to cancer either through cell size or cell number. Therefore, effective tests of Peto’s paradox cannot be performed on animals of different sizes because of the confounding effect of body mass on both cell size/number and metabolic rate. Reasonably chosen organismal models will enable us to investigate the effect of cell size/number on a scale comparable to studies based on small vs. large animals. The possible molecular mechanisms of tumor suppression suggest that not a single mutation but rather a complex adaptation may represent species-specific cancer resistance [12]. To understand these changes in the context of evolutionary fitness, a broader ecological perspective is still needed. Although it seems that cancer remains unavoidable in the living world, consistent and unambiguous approaches to tumor biology may allow us to develop new methods of cancer treatment and prevention.

## Supplementary Information

**Additional file 1.** Original data base for the red blood cells volume after Savage et al. (2007) used for the heterogeneity of the slopes analysis. Records marked with the red color indicate original errors in body mass.

**Additional file 2.** Data base for the body mass, basal metabolic rate and erythrocytes area of mammals used for the conventional and phylogeny-informed analyzes.

**Additional file 3.** Data base for the body mass, resting metabolic rate and erythrocytes area of birds used for the conventional and phylogeny-informed analyzes.

**Additional file 4.** Material and Methods for the conventional and phylogeny-informed analyzes of the correlations of erythrocytes area with body mass in mammals and birds.

## Data Availability

The datasets used and/or analyzed during the current study are available in supplementary materials.

## Abbreviations

BMR: Basal metabolic rate
CS: Cell size
RBC: Red blood cells
GH: Growth hormone
IGF: Insulin-like growth hormone
mTOR: Mechanistic target of rapamycin
Akt: Protein kinase B (former PKB)
PI3K: Phosphoinositide 3-kinase
tNOX: Tumor-associated NADH oxidase
