# Supplementary material for: Cell size, body size and Peto’s paradox
Source: BMC Ecol Evol. 2022 Dec 13;22:142. doi: 10.1186/s12862-022-02096-5 (PMC9746147; doi:10.1186/s12862-022-02096-5)
Supplement: Supplementary file 4 — Additional file 4. Material and Methods for the conventional and phylogeny-informed analyzes of the correlations of erythrocytes area with body mass in mammals and birds. [file 12862_2022_2096_MOESM4_ESM.docx]

**Material and methods**

Based on the available literature, data for the basal metabolic rate (BMR, mammals)/ resting metabolic rate (RMR, birds), erythrocyte area (EA), and body weight (BM) were collected (Additional file 2 and 3). From among the selected records, for further analyzes those, that were characterized by a consistent measurement methodology were used only. The considered values ​​of BMR/RMR (ml O_2_/h - mammals, wats (W) - birds;) were consistent with the standard definition of basal metabolic rate. In case, where the available records were expressed in other units, they were converted to the above-mentioned ones. If available, the size of the erythrocytes referred to their area (µm^2^) was used directly from the source data; other case, it was calculated from the cell diameter, according to the formula of the circle area (mammals) or the ellipse area (birds and mammals family *Camelidae*).

EA vs. BM and BMR/RMR vs. EA were tested, both with conventional methods (Pearson’s correlation) and with the use of phylogeny-informed analysis. As both, the metabolic rate and the size of cells are functions of body mass, BMR/RMR was expressed as residual MR values ​​(weight corrected, RSD). For phylogeny-informed analysis, phylogenetic super-trees were constructed for the selected records (see below). Species of unresolved or uncertain origins (so-called polytomies) were not taken into account when constructing the dendrograms. PDAP program (Phenotypic Diversity Analysis Program, version 6.0 2002) was used for calculating of Phylogenetically Independent Contrasts (PIC). The length of the tree branch was transformed using the Nee method (Purvis 1995). The calculated contrasts were standardized and positivized (Garland et al. 1999, Garland et al. 2005), and then recalculated using classical methods (regression, correlation). The correctness of standardization was checked by testing the significance of a juxtaposition of standardized contrasts with their standard deviation (Garland et al. 1999).

Fig. 1. Phylogenetic supertrees of mammals used for the phylogeny-informed analysis (see Supplementary Materials: Materials and Methods). Branches length were transformed according to the Nee’s method (Purvis 1995). A – general phylogenetic tree representing major systematic groups and orders in mammals; B – Marsupialia; C – Afrotheria; D –Xenarthra; E – Eulipotyhla (Erinaceomorpha i Soricomorpha); F – Chiroptera; G – Carnivora + Pholidota; H – Perrisodactyla + Artiodactyla; I – Rodentia; I1 – Rodentia - Lagomorpha; I2 – Rodentia - Sciuromorpha; I3 – Rodentia - Hystricomorpha; I4 – Rodentia - Castorimorpha; J –Primates + Scandetia;

A

B

C

**D**

**E**

F

G

H

I

I1

I2

I3

I4

J

Fig. 2. Phylogenetic supertrees of birds used for the phylogeny-informed analysis (see Supplementary Materials: Materials and Methods). Branches length were transformed according to the Nee’s method (Purvis 1995). A – general phylogenetic tree representing major systematic groups and orders in birds; B – Paleognathes; C – Galloanserae (Galliformes + Anseriformes); D - Metaves (Columbiformes + Caprimulgiformes + Apodiformes); E – Coronaves I – Charadriformes; F – Coronaves I – Ciconiformes + Pelecaniformes + Procellariformes + Sphenisciformes; G – Coronaves I – Gruiformes + Cuculiformes + Podicipediformes; H – Coronaves II – Falconiformes; I – Coronaves II – Strigiformes; J – Coronaves II – Colliformes + Trogoniformes + Coraciformes + Piciformes; K – Coronaves II – Psittaciformes; L – Coronaves II – Passeriformes; L1 – Coronaves II – Passeriformes – Passeroidea;

A

B

C

D

E

F

G

H

I

J

K

L

L1

**References for Fig. 1SM.**

Agnarsson I., May-Collado L.J. 2008. The phylogeny of *Cetartiodactyla*: The importance of dense taxon sampling, missing data, and the remarkable promise of cytochrome b to provide reliable species-level phylogenies. Mol. Phylo. Evol. 48: 964–985.

Almeida F.C., Bonvicino C.R., Cordeiro-Estrela P. 2007. Phylogeny and temporal diversiffcation of *Calomys* (*Rodentia*, *Sigmodontinae*): Implications for the biogeography of an endemic genus of the open/dry biomes of South America. Mol. Phylo. Evol. 42: 449–466.

Alves P.C., Ferrand N., Suchentrunk F., Harris D.J. 2003. Ancient introgression of *Lepus timidus* mtDNA into *L. granatensis* and *L. europaeus* in the Iberian Peninsula. Mol. Phylo. Evol. 27: 70–80.

Amrine-Madsen H., Koepfli K.P, Wayne R.K., Springer M.S. 2003. A new phylogenetic marker, apolipoprotein B, provides compelling evidence for eutherian relationships. Mol. Phylo. Evol. 28: 225–240.

Arnason U., Adegoke J.A., Bodin K., Born E.W., Esa Y.B., Gullberg A., Nilsson M., Short R.V., Xu X., Janke A. 2002. Mammalian mitogenomic relationships and the root of the eutherian tree. PNAS 11: 8151–8156.

Arnason U., Gullberg A., Janke A., Kullberg M., Lehman N., Petrov E.A., Väinölä R. 2006. Pinniped phylogeny and a new hypothesis for their origin and dispersal. Mol. Phylo. Evol. 41: 345–354.

Asher R.J., Hofreiter M. 2006.Tenrec Phylogeny and the Noninvasive Extraction of Nuclear DNA. Syst. Biol*.* 55(2):181–194

Asher R.J. 2007. A web-database of mammalian morphology and a reanalysis of placental phylogeny. BMC Evolutionary Biology 7:108.

Bannikova A.A., Matveev V.A., Kramerov D.A. 2002. Using Inter-SINE–PCR to Study Mammalian Phylogeny. Russ. J. Gen. 38: 714–724.

Bannikovaa A.A., Lavrenchenkob L.A., Kramerov D.A. 2005. Phylogenetic relationships between Afrotropical and Palaearctic Crocidura species inferred from Inter-SINE-PCR. Biochem. Sys. Ecol. 33: 45–59.

Bardeleben C., Moore R.L., Wayne R.K. 2005. A molecular phylogeny of the *Canidae* based on six nuclear loci. Mol. Phylo. Evol. 37: 815–831.

Beck R.M.D., Bininda-Emonds O.R.P., Cardillo M., Liu F.R., Purvis A. 2006. A higher-level MRP supertree of placental mammals. BMC Evolutionary Biology 6: 93-99.

Bradley R.D., Durish N.D., Rogers D.S., Miller J.R., Engstrom M.D., Kilpatrick C.W. 2007. Toward a molecular phylogeny for *Peromyscus*: Evidence from mitochondrial cytochrome-b sequences. J. Mamm. 88(5): 1146–1159.

Cardillo M., Bininda-Emonds O.R.P., Boakes E., Purvis A. 2004. A species-level phylogenetic supertree of marsupials. J. Zool. 264: 11–31.

Chevret P., Dobigny G. 2005. Systematics and evolution of the subfamily *Gerbillinae* (*Mammalia*, *Rodentia*, *Muridae*). Mol. Phylo. Evol. 35: 674–688.

Colangelo P., Corti M., Verheyen E., Annesi F., Oguge N., Makundi R.H., Verheyen W. 2005. Mitochondrial phylogeny reveals differential modes of chromosomal evolution in the genus *Tatera* (*Rodentia*: *Gerbillinae*) in Africa. Mol. Phylo. Evol. 35: 556–568.

Cox P.G. 2006 Character evolution in the orbital region of the Afrotheria. J. Zool. 269: 514-526.

Cutrera A.P., Lacey E.A. 2007. Trans-species polymorphism and evidence of selectionon class II MHC loci in tuco-tucos (*Rodentia*: *Ctenomyidae*). Immunogenetics 59: 937–948.

Da´ Valos L.M., Jansa S.A. 2004. Phylogeny of the lonchophyllini (*Chiroptera*: *Phyllostomidae*). J. Mamm. 85(3): 404–413.

Delpero M., Masters J.C., Cervella P., Crovella S., Ardito G., Rumpler Y. 2001. Phylogenetic relationships among the Malagasy lemuriforms (*Primates*: *Strepsirrhini*) as indicatedby mitochondrial sequence data from the 12S rRNA gene. Zool. J. Linn. Soc. 133: 83–103.

Douady Ch.J., Catzeflis F.O., Raman J., Springer M.S., Stanhope M.J. 2003. The Sahara as a vicariant agent, and the role of Miocene climatic events, in the diversification of the mammalian order *Macroscelidea* (elephant shrews). PNAS 100: 8325–8330.

Douady Ch.J., Scally M., Springer M.S., Stanhope M.J. 2004. ‘‘Lipotyphlan’’ phylogeny based on the growth hormone receptor gene: a reanalysis. Mol. Phylo. Evol. 30: 778–788.

Dubey S., Antonina M., Denys Ch., Vogel P. 2007. Use of phylogeny to resolve the taxonomy of the widespread and highly polymorphic African giant shrews (*Crocidura olivieri* group, *Crocidurinae*, *Mammalia*). Zoology 110: 48–57.

Dubey S., Salamin N., Ruedi M., Barričre P., Colyn M., Vogel P. 2008. Biogeographic origin and radiation of the Old World crocidurine shrews (*Mammalia*: *Soricidae*) inferred from mitochondrial and nuclear genes. Mol. Phylo. Evol. 48: 953–963.

Fulton T.L., Strobeck C. 2006. Molecular phylogeny of the *Arctoidea* (*Carnivora*): Effect of missing data on supertree and supermatrix analyses of multiple gene data sets. Mol. Phylo. Evol. 41: 165–181

Galewski T., Tilak M., Sanchez S., Chevret P.,Paradis E., Douzery E.J.P. 2006. The evolutionary radiation of *Arvicolinae* rodents (voles and lemmings): relative contribution of nuclear and mitochondrial DNA phylogenies. BMC Evolutionary Biology 6:80.

Giannini N.P., Almeida F.C., Simmons N.B., Helgen K.M. 2008. The systematic position of *Pteropus leucopterus* and its bearing on the monophyly and relationships of *Pteropus* (*Chiroptera*: *Pteropodidae*). *Acta Chiropterologica* 10(1): 11–20.

Gilbert C., Ropiquet A., Hassanin A. 2006. Mitochondrial and nuclear phylogenies of *Cervidae* (*Mammalia*, *Ruminantia*): Systematics, morphology, and biogeography. Mol. Phylo.Evol. 40: 101–117.

Hafner J.C., Light J.E., Hafner D.J., Hafner M.S., Reddington E., Rogers D.S., Riddle B.R. 2007. Basal clades and molecular systematics of *Heteromyid* rodents. J. Mamm. 88(5): 1129–1145.

Jansa S.A., Weksler M. 2004. Phylogeny of muroid rodents: relationships within and among major lineages as determined by IRBP gene sequences. Mol. Phylo. Evol. 31: 256–276.

Koito T., Kubokawa K., Tanabe S., Miyazaki N. 2010. Phylogenetic analyses in cetacean species of the family *Delphinidae* using a short wavelength sensitive opsin gene sequence. Fish Sci. 76:571–576.

Lecompte E., Aplin K., Denys Ch., Catzeflis F., Chades M., Chevret P. 2008. Phylogeny and biogeography of African *Murinae* based on mitochondrial and nuclear gene sequences, with a new tribal classification of the subfamily. BMC Evolutionary Biology 8:199.

Liu Fu-Guo R., Miyamoto M.M., Freire N.P., Ong P.Q., Tennant M.R., Young T.S., Gugel K.F. 2001. Molecular and morphological supertrees for eutherian (Placental) mammals. Science 291: 1786-1789.

Masters J.C. 2007. Taking phylogenetics beyond pattern analysis: Can models of genome dynamics guide predictions about homoplasy in morphological and behavioral data sets? J. Hum. Evol. 52: 522-535.

Matoc M.D., Shurtli Q.R., Feldman C.R. 2007. Phylogenetics of the woodrat genus *Neotoma* (*Rodentia*: *Muridae*): Implications for the evolution of phenotypic variation in male external genitalia. Mol. Phylo. Evo. 42: 637–652.

McNab B.K. 2003. Standard energetics of phyllostomid bats: the inadequacies of phylogenetic-contrast analyses. Comp. Biochem. Physiol. 135A: 357–368.

Mercer J.M., Roth V.L. 2003. The effects of cenozoic global change on squirrel phylogeny. Science 299: 1568-1571.

Motokawa M. 2004. Phylogenetic relationships within the family *Talpidae* (*Mammalia*: *Insectivora*). J. Zool. 263: 147–157.

Nishihara H., SattaY., Nikaido M., Thewissen J.G.M., Stanhope M.J., Okada N. 2005. A retroposon analysis of Afrotherian phylogeny. Mol. Biol. Evol. 22(9): 1823–1833.

Nishihara H., Hasegawa M., Okada N. 2006. *Pegasoferae*, an unexpected mammalian clade revealed by tracking ancient retroposon insertions. PNAS 27: 9929–9934.

Ohdachi S.D., Hasegawa M., Iwasa M.A., Vogel P., Oshida T., Lin L.-K, Abe H. 2006. Molecular phylogenetics of soricid shrews (*Mammalia*) based on mitochondrial cytochrome b gene sequences: with special reference to the *Soricinae*. J. Zool. 270: 177–191.

Patou M-L., Debruyne R., Jennings A.P., Zubaid A., Rovie-Ryan J.J., Veron G. 2008. Phylogenetic relationships of the Asian palm civets (*Hemigalinae* & *Paradoxurinae*, *Viverridae*, *Carnivora*). Mol. Phylo. Evol. 47: 883–892.

Riddle B.R., Hafner D.J., Alexander L.F. 2000. Comparative phylogeography of Baileys’ pocket mouse (*Chaetodipus baileyi*) and the *Peromyscus eremicus* species group: historical vicariance of the Baja California Peninsular desert. Mol. Phylo. Evol. 17: 161–172

Rodriguez-Serranoa E., Hernándeza C.E., Palma R.E. 2007. A new record and an evaluation of the phylogenetic relationships of *Abrothrix olivaceus* markhami (*Rodentia*: *Sigmodontinae*). Mamm. Biol. 146: 210-218.

Rowe K.C., Reno M.L., Richmond D.M., Adkins R.M., Steppan S.J. 2008. Pliocene colonization and adaptive radiations in Australia and New Guinea (Sahul): Multilocus systematics of the old endemic rodents (*Muroidea*: *Murinae*). Mol. Phylo. Evol. 47: 84–101.

Shelley E.L., Blumstein D.T. 2004. The evolution of vocal alarm communication in rodents. Behavioral Ecology 16: 169-177.

Stadelmann B., Lin L.-K., Kunz T.H., Ruedi M. 2007. Molecular phylogeny of New World *Myotis* (*Chiroptera*, *Vespertilionidae*) inferred from mitochondrial and nuclear DNA genes. Mol. Phylo. Evol. 43: 32–48.

Steppan S.J., Storz B.L., Hoffmann R.S. 2004. Nuclear DNA phylogeny of the squirrels (*Mammalia*: *Rodentia*) and the evolution of arboreality from c-myc and RAG1. Mol. Phylo. Evol. 30: 703–719.

Steppan S.J., Ramirez O., Banbury J., Huchon D., Pacheco V., Walker L.I., Spotorno A.E. 2007. A molecular reappraisal of the systematics of the leaf-eared Mice *Phyllotis* and their relatives. W: *The Quintessential Naturalist: Honoring the Life and Legacy of Oliver P. Pearson*, Kelt D.A., E.P. Lessa, Salazar-Bravo J., Patton J.L. (wyd.), University of California Publications in Zoology 134: 799-826.

Sylvain D.S., Salamin N., Ohdachi S.D., Barrie`re P., Vogel P. 2007. Molecular phylogenetics of shrews (Mammalia: *Soricidae*) reveal timing of transcontinental colonizations. Mol. Phylo. Evol. 44: 126–137.

Weksler M. 2003. Phylogeny of Neotropical oryzomyine rodents (*Muridae*: *Sigmodontinae*) based on the nuclear IRBP exon. Mol. Phylo. Evol. 29: 331–349.

Veron G., Colyn M., Dunham A.E., Taylor P., Gaubert P. 2004. Molecular systematics and origin of sociality in mongooses (*Herpestidae*, *Carnivora*). Mol. Phylo. Evol. 30: 582-598.

Yasuda S.M., Sato J., Vogel P., Suzuki H. 2007. Phylogenetic relationships and divergence times among dormice (*Rodentia*, *Gliridae*) based on three nuclear genes. *Zoologica Scripta* 36: 537–546.

**References for Fig. 2SM.**

Alström P., Ericson P.G.P., Olsson U., Sundberg P. 2006. Phylogeny and classification of the avian superfamily *Sylvioidea*. Mol. Phylo. Evol. 38: 381–397.

Barrowclough G.F., Groth J.G, Mertz L.A. 2006. The RAG-1 exon in the avian order Caprimulgiformes: Phylogeny, heterozygosity, and base composition. Mol. Phylo. Evol. 41: 238–248.

Bonaccorso E., Petersom A.T. 2007. A multilocus phylogeny of New World jay genera. Mol. Phylo. Evol. 42 (2007) 467–476.

Bridgea E.S., Jones A.W., Baker A.J. 2005. A phylogenetic framework for the terns (Sternini) inferred from mtDNA sequences: implications for taxonomy and plumage evolution. Mol. Phylo. Evol. 35: 459–469.

Bried J.L, Pontier D., Jouventin P. 2003. Mate fidelity in monogamous birds: a re-examination of the Procellariiformes. Animal Behaviour. 65: 235–246.

Carson R.J., Spicer G.S. 2003. A phylogenetic analysis of the emberizid sparrows based on three mitochondrial genes. Mol. Phylo. Evol. 29: 43–57.

Chesser R.T. 2004. Molecular systematics of New World suboscine birds. Mol. Phylo. Evol. 32: 11–24.

Chu P.C. 1998. A Phylogeny of the Gulls (Aves: Larinae) Inferred from Osteological and Integumentary Characters. Cladistics 14: 1–43.

Chu P.C. 2002. Morphological Test of the Monophyly of the Cardueline Finches (Aves: Fringillidae, Carduelinae). Cladistics 18: 279–312.

Cibois A., Cracraft J. 2004. Assessing the passerine “Tapestry”: phylogenetic relationships of the *Muscicapoidea* inferred from nuclear DNA sequences. Mol. Phylo. Evol. 32: 264–273.

Cicero C., Johnson N.K. 2002. Phylogeny and character evolution in the *Empidonax* group of tyrant flycatchers (Aves: *Tyrannidae*): a test of W. E. Lanyon’s hypothesis using mtDNA sequences. Mol. Phylo. Evol. 22: 289–302.

Clarke J.A., Ksepka D.T., Stucchi M., Urbina M., Gianninig N., Bertelli S., Narváez Y., Boyd C.A. 2007. Paleogene equatorial penguins challenge the proposed relationship between biogeography, diversity, and Cenozoic climate change. PNAS 107: 11545-11550.

Crowe T.M, Bloomer P., Randi E., Lucchini V., Kimball R., Braun E., Groth J.G. 2006. Supra-generic cladistics of landfowl (Order Galliformes). A. Zool. Sinica 52(Suppl): 358–361.

Cubo J., Arthur W. 2001. Patterns of correlated character evolution in flightless birds: a phylogenetic approach. Evol. Ecol. 14: 693-702.

Dai Ch, Chen K., Zhang R., Yang X., Yin Z., Tian H., Zhang Z., Hu Y., Lei F. 2010. Molecular phylogenetic analysis among species of *Paridae*, *Remizidae* and *Aegithalos* based on mtDNA sequences of COI and cyt b. Chinese Birds 1(2):112–123.

De Kloet R.S., De Kloet S.R. 2005. The evolution of the spindlin gene in birds: Sequence analysis of an intron of the spindlin W and Z gene reveals four major divisions of the *Psittaciformes*. Mol. Phylo. Evol. 36: 706–721.

DeFilippis V.R., Moore V.S. 2000. Resolution of phylogenetic relationships among recently evolved species as a function of amount of DNA sequence: An empirical study based on woodpeckers (Aves: *Picidae*). Mol. Phylo. Evol. 16: 143–160.

Dimcheff D.E., Drovetski S.V., Mindell D.P. 2002. Phylogeny of *Tetraoninae* and other galliform birds using mitochondrial 12S and ND2 genes. Mol. Phylo. Evol. 24: 203–215.

Donne-Gouss C., Laudet V., Hänni C. 2002. A molecular phylogeny of *anseriformes* based on mitochondrial DNA analysis. Mol. Phylo. Evol. 23: 339–356.

Driskella A.C., Christidis L. 2004. Phylogeny and evolution of the Australo-Papuan honeyeaters (*Passeriformes*, *Meliphagidae*). Mol. Phylo. Evol. 31: 943–960.

Ericson P.G.P., Anderson C.L., Britton T., Elzanowski A., Johansson U.S., Kallersjo M., Ohlson J.I., Parsons T.J., Zuccon D., Mayr G. 2006. Diversification of Neoaves: integration of molecular sequence data and fossils. Biol. Lett. 2: 543–547.

Ericson P.G.P., Zuccon D., Ohlson J.I., Johansson U.S., Alvarenga H., Prum R.O. 2006. Higher-level phylogeny and morphological evolution of tyrant Flycatchers, cotingas, manakins, and their allies (Aves: *Tyrannida*). Mol. Phylo. Evol. 40: 471–483.

Ericsona P.G.P., Johanssona U.S. 2003. Phylogeny of *Passerida* (Aves: Passeriformes) based on nuclear and mitochondrial sequence data. Mol. Phylo. Evol. 29: 126–138.

Fain M.G., Houde P. 2007. Multilocus perspectives on the monophyly order Charadriformes (Aves). BMC 7:35.

Fain M.G., Krajewski C., Houde P. 2007. Phylogeny of “core Gruiformes” (Aves: Grues) and resolution of the Limpkin–Sungrebe problem. Mol. Phylo. Evol. 43: 515–529.

Fuchs J., Ohlson J.I., Ericson P.G.P., Pasquet E. 2006. Molecular phylogeny and biogeographic history of the piculets (*Piciformes*: *Picumninae*). J. Avian Biol. 37: 487-496.

Geffen E., Yom-Tov Y. 2001. Factors affecting the rates of intraspecific nest parasitism among Anseriformes and Galliformes. Animal Behaviour 62: 1027–1038.

Gerwin J.A., Zink R.M. 1998. Phylogenetic patterns in the *Trochilidae*. The Auk 115(1): 105-118.

Gibb G.C., Kardailsky O., Kimball R.T., Braun E.L., Penny D. 2007. Mitochondrial genomes and avian phylogeny: Complex characters and resolvability without explosive radiations Mol. Biol. Evol. 24:269–280.

Haring E., Gamauf A., Kryukov A. 2007. Phylogeographic patterns in widespread corvid birds. Mol. Phylo. Evol. 45: 840–862.

Heidrich P., Amengual J., Wink M. 1998. Phylogenetic relationships in Mediterranean and North Atlantic shearwaters (Aves: *Procellariidate*) based on nucleotide of mtDNA. Biochem. Syst. Ecol. 26: 145 – 170.

Irestedt M., Fjelds J., Johansson U.S., Ericssona P.G.P. 2002. Systematic relationships and biogeography of the tracheophone suboscines (Aves: Passeriformes). Mol. Phylo. Evol. 23: 499–512.

Kennedy M., Gray R.D., Spencer H.G. 2000. The Phylogenetic Relationships of the Shags and Cormorants: Can sequence data resolve a disagreement between behavior and morphology? Mol. Phylo. Evol. 17: 345–359.

Kimball R.T., Braun E.L, Zwartjes P.W., Crowe T.M., Ligon J.D. 1999. A molecular phylogeny of the pheasants and partridges suggests that these lineages are not monophyletic. Mol. Phylo. Evol. 11: 38–54.

Klicka J., Burns K., Spellman G.M. 2007. Defining a monophyletic *Cardinalini*: A molecular perspective. Mol. Phylo. Evol. 45: 1014–1032.

Klicka J., Voelkerb G., Spellmana G.M. 2005. A molecular phylogenetic analysis of the “true thrushes” (Aves: Turdinae). Mol. Phylo. Evol. 34: 486–500.

Larsen C., Speed M., Harvey N., Noyes H.A. 2007. A molecular phylogeny of the nightjars (Aves: *Caprimulgidae*) suggests extensive conservation of primitive morphological traits across multiple lineages. Mol. Phylo. Evol. 42: 789–796.

Lei X., Yin Z., Lian Z., Chen C., Dai Ch., Krištín A., Lei F. 2010. Phylogenetic relationships of some *Sylviidae* species based on complete mtDNA cyt b and partial COI sequence data. Chinese Birds 1(3):175–187.

Lerner H.R.L., Mindell D.P. 2005. Phylogeny of eagles, Old World vultures, and other *Accipitridae* based on nuclear and mitochondrial DNA. Mol. Phylo. Evol. 37: 327–346.

Livezey B.C. 1998. A phylogenetic analysis of the Gruiformes (Aves) based on morphological characters, with an emphasis on the rails (*Rallidae*). Phil.Trans. R. Soc. Lond. B 353: 2077-2151.

Lovette I.J., Rubenstein D.R. 2007. A comprehensive molecular phylogeny of the starlings (Aves: *Sturnidae*) and mockingbirds (Aves: *Mimidae*): Congruent mtDNA and nuclear trees for a cosmopolitan avian radiation. Mol. Phylo. Evol. 44. 1031–1056.

Lovette I.J., McCleery B.V., Talaba A.L., Rubenstein D.R. 2008. A complete species-level molecular phylogeny for the “Eurasian” starlings (*Sturnidae: Sturnus, Acridotheres*, and allies): Recent diversification in a highly social and dispersive avian group. Mol. Phylo. Evol. 47: 251–260.

Mayr G. 2003. On the phylogenetic relationships of trogons (Aves, *Trogonidae*). J. Avian Biol. 34: 81–88.

Mayr G., Clarke J. 2003. The deep divergences of neornithine birds: a phylogenetic analysis of morphological characters. Cladistics 19: 527–553.

McKechnie A.E., Freckleton R.P., Jetz W. 2006. Phenotypic plasticity in the scaling of avian basal metabolic rate. Proc. R. Soc. B. 273: 931–937.

Monteros A.E. 2000. Higher-Level Phylogeny of Trogoniformes. Mol. Phylo. Evol. 14: 20–34.

Morgan-Richards M., Trewick S.A., Bartosch-Härlid A., Kardailsky O., Phillips M.J., McLenachan M.A., Chubb A.L. 2004. New nuclear evidence for the oldest divergence among neognath birds: the phylogenetic utility of zenk. Mol. Phylo. Evol. 30: 140–151.

Moyle R.G. 2004. Phylogenetics of barbets (Aves: *Piciformes*) based on nuclear and mitochondrial DNA sequence data. Mol. Phylo. Evol. 30: 187–200.

Moyle R.G., Marks B.D. 2006. Phylogenetic relationships of the bulbuls (Aves: *Pycnonotidae*) based on mitochondrial and nuclear DNA sequence data. Mol. Phylo. Evol. 40: 687–695.

Ohlson J.I., Prum R.O., Ericson P.G.P. 2007. A molecular phylogeny of the cotingas (Aves: *Cotingidae*). Mol. Phylo. Evol. 42: 25–37.

Omland K.E., Lanyon S.M., Fritz S.J. 1999. A molecular phylogeny of the NewWorld orioles (*Icterus*): the importance of dense taxon sampling. Mol. Phylo. Evol. 12: 224–239.

Overton L.C., Rhoads D.D. 2006. Molecular phylogenetic relationships of *Xiphidiopicus* *percussus*, *Melanerpes*, and *Sphyrapicus* (Aves: *Picidae*) based on cytochrome b sequence. Mol. Phylo. Evol. 41: 288–294.

Pereira S.L., Baker A.J. 2006. A molecular timescale for galliform birds accounting for uncertainty in time estimates and heterogeneity of rates of DNA substitutions across lineages and sites. Mol. Phylo. Evol. 38: 499–509.

Pereira S.L., Baker A.J. 2008. DNA evidence for a Paleocene origin of the *Alcidae* (Aves: Charadriiformes) in the Pacific and multiple dispersals across northern oceans. Mol. Phylo. Evol. 46: 430–445.

Riesing M.J., Kruckenhauser L., Gamauf A., Haring E. 2003. Molecular phylogeny of the genus *Buteo* (Aves: *Accipitridae*) based on mitochondrial marker sequences. Mol. Phylo. Evol. 27: 328–342.

Russello M.A., Amato G. 2004. A molecular phylogeny of *Amazona*: implications for Neotropical parrot biogeography, taxonomy, and conservation. Mol. Phylo. Evol. 30: 421–437.

Salzburger W., Martens J., Sturmbauer C. 2002. Paraphyly of the Blue Tit (*Parus caeruleus*) suggested from cytochrome b sequences. Mol. Phylo. Evol. 24: 19–25.

Saunders M.A., Edwards S.V. 2000. Dynamics and phylogenetic implications of mtDNA control region sequences in New World jays (Aves: *Corvidae*). J. Mol. Evol. 51: 97–109.

Sheldon F.H., Jones C.E., McCracken K.G. 2000. Relative patterns and rates of evolution in heron nuclear and mitochondrial DNA. Mol. Biol. Evol*.* 17: 437–450.

Sheldon F.H., Whittingham L.A., Winkler D.W. 1999. A Comparison of Cytochrome b and DNA Hybridization Data Bearing on the Phylogeny of Swallows (Aves: *Hirundinidae*). Mol. Phylo. Evol. 11: 320–331.

Slikas B. 1997. Phylogeny of the Avian Family *Ciconiidae* (Storks) Based on Cytochrome *b* Sequences and DNA–DNA Hybridization Distances. Mol. Phylo. Evol. 3: 275–300.

Smith E.J., Shi L., Tu Z. 2005. Gallus gallus aggrecan gene-based phylogenetic analysis of selected avian taxonomic groups. Genetica 124: 23–32.

Spicer G.S., Dunipace L. 2004. Molecular phylogeny of songbirds (Passeriformes) inferred from mitochondrial 16S ribosomal RNA gene sequences. Mol. Phylo. Evol. 30: 325–335.

Thomas G.H., Wills M.A., Székely T. 2004. A supertree approach to shorebird phylogeny. BMC 4:28.

Van der Meij M.A.A., de Bakker M.A.G., Bout R.G. 2005. Phylogenetic relationships of Wnches and allies based on nuclear and mitochondrial DNA. Mol. Phylo. Evol. 34: 97–105.

Voelker G., Spellman G.M. 2004. Nuclear and mitochondrial DNA evidence of polyphyly in the avian superfamily *Muscicapoidea*. Mol. Phylo. Evol. 30: 386–394.

Voelker G., Rohwer S., Bowie R.C.K., Outlaw D.C. 2007. Molecular systematics of a speciose, cosmopolitan songbird genus: Defining the limits of, and relationships among, the Turdus thrushes. Mol. Phylo. Evol. 42: 422–434.

Wink M., Heidrich P., Fentzloffs C. 1996. A mtDNA phylogeny of sea eagles (genus *Hahaeetus)* based on nucleotide sequences of the cytochrome b-gene. Bio Sys. Ecol. 718: 783-791.

Woog F., Wink M., Rastegar-Pouyani E., Gonzalez J., Helm B. 2008. Distinct taxonomic position of the Madagascar stonechat (*Saxicola* *torquatus* *sibilla*) revealed by nucleotide sequences of mitochondrial DNA. J. Ornithol. 149: 423–430.

Yuri T., Mindell D.P. 2002. Molecular phylogenetic analysis of *Fringillidae*, “New World nine-primaried oscines” (Aves: Passeriformes). Mol. Phylo. Evol. 23: 229–243.
